# Atrazine and Cancer Incidence Among Pesticide Applicators in the Agricultural Health Study (1994–2007)

**DOI:** 10.1289/ehp.1103561

**Published:** 2011-05-27

**Authors:** Laura E. Beane Freeman, Jennifer A. Rusiecki, Jane A. Hoppin, Jay H. Lubin, Stella Koutros, Gabriella Andreotti, Shelia Hoar Zahm, Cynthia J. Hines, Joseph B. Coble, Francesco Barone-Adesi, Jennifer Sloan, Dale P. Sandler, Aaron Blair, Michael C.R. Alavanja

**Affiliations:** 1 Division of Cancer, Epidemiology, and Genetics, National Cancer Institute, National Institutes of Health, Department of Health and Human Services, Bethesda, Maryland, USA; 2 Department of Preventive Medicine and Biometrics, Uniformed Services University of the Health Sciences, Bethesda, Maryland, USA; 3 Epidemiology Branch, National Institute of Environmental Health Sciences, National Institutes of Health, Department of Health and Human Services, Research Triangle Park, North Carolina, USA; 4 National Institute for Occupational Safety and Health, Cincinnati, Ohio, USA

**Keywords:** agriculture, atrazine, cancer, cohort study, epidemiology, pesticide

## Abstract

Background: Atrazine is a triazine herbicide used widely in the United States. Although it is an animal carcinogen, the mechanism in rodents does not appear to operate in humans. Few epidemiologic studies have provided evidence for an association.

Methods: The Agricultural Health Study (AHS) is a prospective cohort that includes 57,310 licensed pesticide applicators. In this report, we extend a previous AHS analysis of cancer risk associated with self-reported atrazine use with six additional years of follow-up and more than twice as many cancer cases. Using Poisson regression, we calculated relative risk estimates and 95% confidence intervals for lifetime use of atrazine and intensity-weighted lifetime days, which accounts for factors that impact exposure.

Results: Overall, 36,357 (68%) of applicators reported using atrazine, among whom there were 3,146 cancer cases. There was no increase among atrazine users in overall cancer risk or at most cancer sites in the higher exposure categories compared with the lowest. Based on 29 exposed cases of thyroid cancer, there was a statistically significant risk in the second and fourth quartiles of intensity-weighted lifetime days. There was a similar pattern for lifetime days, but neither the risk estimates nor the trend were statistically significant and for neither metric was the trend monotonic.

Conclusions: Overall, there was no consistent evidence of an association between atrazine use and any cancer site. There was a suggestion of increased risk of thyroid cancer, but these results are based on relatively small numbers and minimal supporting evidence.

Atrazine (6-chloro-*N*-ethyl-*N´*-(1-methylethyl)-1,3,5-triazine-2,4-diamine) is a triazine herbicide widely used in the production of corn and other agricultural crops in the United States. Atrazine loss from the soil to surface water is estimated at 1% per year (Goolsby et al. 1991) and may be transported to areas as far as 1,000 km from the site of use via atmospheric transport and deposition through precipitation (Mast et al. 2007; Thurman and Cromwell 2000). Several studies have suggested that atrazine is an endocrine disruptor in multiple species, including amphibians (Hayes et al. 2002, 2010), fish (Moore and Waring 1998), and at high exposure levels in birds (Matsushita 2006) and rodents (Eldridge et al. 1999; Friedman 2002; Stoker et al. 2000). Some studies have suggested that the lower exposure levels that occur in the environment may be insufficient to affect reproduction in fish, amphibians, or reptiles (Solomon et al. 2008). In 2004, the European Union banned the use of atrazine because of its contamination of water sources (European Commission 2004). The International Agency for Research on Cancer (IARC) lists atrazine as not classifiable as to its carcinogenicity to humans (Group 3), although it did determine that there is sufficient evidence that it causes cancer in rodents (IARC 1999). The U.S. Environmental Protection Agency (EPA) is currently reviewing its registration decision on atrazine (U.S. EPA 2009).

A few epidemiologic studies have evaluated the relationship between atrazine and cancer. Case–control studies have shown weak associations with non-Hodgkin lymphoma (NHL) (De Roos et al. 2003; Hoar Zahm et al. 1993; Zahm et al. 1993) and have suggested an increased risk of ovarian (Donna et al. 1989) and prostate cancers (MacLennan et al. 2002). No associations have been observed with leukemia, Hodgkin lymphoma, multiple myeloma (Brown et al. 1993), soft-tissue sarcoma (Hoar et al. 1986), or colon cancer (Hoar et al. 1985).

Within the Agricultural Health Study (AHS), a large, prospective cohort of licensed pesticide applicators and their spouses, a previous analysis of cancer and atrazine use among pesticide applicators found suggestive associations with NHL, multiple myeloma, and cancers of the bladder and lung, but none were statistically significant (Rusiecki et al. 2004). The current study updates the previous analysis with an additional 6 years of cancer incidence and follow-up data, including more than twice as many incident cancer cases (*n* = 3,146), 1,785 of which are additional cases since the previous publication.

## Methods

*Cohort description.* The AHS is a prospective cohort study that includes 57,310 licensed pesticide applicators in Iowa and North Carolina. Applicators were recruited and enrolled in the study during 1993–1997 when they obtained or renewed their licenses to apply restricted use pesticides. In North Carolina, only private applicators, who are primarily farmers, were recruited, whereas in Iowa, both private and commercial applicators were included. Commercial applicators included persons employed by pest control companies or businesses that use pesticides. Incident cancers were ascertained through linkage to state cancer registries in Iowa and North Carolina. First primary cancers diagnosed from enrollment through 31 December 2007 were included in this analysis. Annually, cohort members were matched to the National Death Index to identify vital status and to current address records of the Internal Revenue Service, motor vehicle registration offices, and pesticide license registries of state agricultural departments to determine whether they continued to reside in Iowa or North Carolina. Follow-up was censored at the time of cancer incidence, death, or movement out of the state. Participants provided informed consent, and the study protocol was approved by the institutional review boards of the National Institutes of Health, the University of Iowa, and other contractors in compliance with all applicable requirements of the United States.

*Exposure assessment.* Use of atrazine and other factors was assessed through the completion of a self-administered questionnaire (available at http://aghealth.nci.nih.gov/questionnaires.html). Participants provided information on their use of 50 common pesticides, pesticide application and mixing methods, repair of pesticide application equipment, and use of personal protective equipment. Participants also reported on potential risk factors such as smoking, alcohol consumption, cancer history of first-degree relatives, diet, selected medical conditions, and demographic information.

Questionnaire data were used to construct two measures of atrazine use. The first metric was lifetime days of use, calculated by multiplying the years of reported atrazine use by the average number of days per year of use. The second exposure metric was intensity-weighted lifetime days of use, calculated by multiplying lifetime days of atrazine use by a measure of exposure intensity based on general handling practices for pesticides. This intensity-weighting algorithm included factors that impacted exposure levels, such as whether the applicator personally mixed pesticides or repaired application equipment, the methods of applying pesticides, and the use and type of personal protective equipment when handling pesticides (Dosemeci et al. 2002). The original intensity-weighting algorithm was based on questionnaire information, expert review of relative exposure levels from published literature on pesticide exposure, and information from the Pesticide Handlers Exposure Database (PHED 1992). We recently modified the weighting factors for the intensity-weighting algorithm, using data from pesticide monitoring studies conducted in subsets of AHS participants (Hines et al. 2008; Thomas et al. 2010a, 2010b). The results from these substudies supported the use of the algorithm to assign exposure intensities but indicated areas where refinement could be made. The updated (v2) algorithm gave more weight to the use of chemically resistant gloves for reducing exposure, increased the score given to broadcast methods of application relative to in-furrow application, decreased the score given to mixing activities relative to the original version of the algorithm, and rescaled the range to include integer values.

*Data analysis.* Of the 57,310 applicators in the AHS, we excluded 2,337 who provided no or insufficient information on atrazine use to calculate lifetime or intensity-weighted lifetime days, 1,044 with a cancer diagnosis before enrollment, and 267 who had missing or zero person-years, leaving 53,662 applicators available for analyses.

We categorized lifetime days of use and intensity-weighted lifetime days of use into nonexposed and quartiles of exposure among users, with categories based on the distribution of all cancer cases combined. We report results for cancer sites with > 20 exposed cases and for breast and ovarian cancer, which were sites with high *a priori* interest because of the hormonal properties of atrazine. Relative risks (RRs) were calculated by Poisson regression using SAS (version 9.1; SAS Institute Inc., Cary, NC). Subjects contributed person-time at risk from year of enrollment (1993–1997) through 31 December 2007, date of first cancer diagnosis or date moved out of the state, whichever was earlier.

Similar to the previous publication, unless otherwise noted, models were adjusted for race, sex, age (< 50, 50–59, 60–69, ≥ 70 years), smoking history (never, ≤ 12 pack-years, > 12 pack-years, regardless of current smoking status), alcohol use (yes/no in preceding 12 months), education (less than high school, high school degree, more than high school), state of residence (Iowa or North Carolina), family history of cancer, applicator type (private or commercial), and ever use of other pesticides most highly correlated with atrazine use [dicamba, cyanazine, metolachlor, trifluralin, 2,4-D (2,4-dichlorophenoxyacetic acid)]. For lung and bladder cancer, we additionally adjusted for smoking with tertiles of pack-years among former and current smokers. For ovarian and breast cancer, because of the small number of cases, confidence limits were calculated in STATA (StataCorp LP, College Station, TX) using exact methods. For all cancer sites evaluated, associations using both the original and the updated versions of the intensity-weighting algorithm were similar; therefore, we present results based on the updated (version 2) algorithm only. All RRs were calculated using two different referent groups, those reporting no use of atrazine and those in the lowest quartile of atrazine use. Applicators in the highest categories of atrazine use were more similar to those in the lowest quartile of use than to nonusers on some characteristics, which might suggest the possible presence of confounding on unmeasured characteristics. Therefore, analyses using the low-exposed group as the referent are reported here. Tests for trend used the midpoint value of each exposure category treated as a continuous variable in Poisson regression models. All tests were two-sided and conducted at the 0.05 alpha level. We performed stratified analyses by state and by applicator type to evaluate potential differences between the two groups. We also stratified on use of chemicals that been reported in a case–control study to have supra-additive effects with atrazine and NHL (carbofuran, diazinon, and alachlor). All data used in this analysis are based on AHS data release P1REL201005.00.

## Results

Selected demographic characteristics and use of correlated pesticides by atrazine use (none, lowest quartile, highest three quartiles) are reported in Table 1. Pesticide applicators in this cohort are overwhelmingly white and male. For many characteristics, such as sex, state, alcohol use, and use of correlated pesticides, applicators in the highest quartiles of use are more similar to applicators in the lowest quartile of use than to the nonexposed. Private pesticide applicators were more likely to have used atrazine than were commercial applicators (70% and 50%, respectively). However, among commercial applicators who reported use of atrazine, the number of days per year and total lifetime days of use were higher than that among private applicators (lifetime days median = 116.0, interquartile range, 49.50–457.25 for commercial; lifetime days median = 56.0, interquartile range, 20.0–178.50 for private applicators).

**Table 1:** Selected characteristics of pesticide applicators, by atrazine use category based on enrollment questionnaire data, 1993–1997 [*n* (%)].

Lifetime days of atrazine use							
Characteristic	No use of atrazine (*n* = 17,305)		Quartile 1 (*n* = 9,523)		Quartiles 2, 3, and 4 (*n* = 26,834)	
Age (years)									
< 50		10,958	(63.3)		5,906	(62.0)		16,526	(61.6)
50–59		3,098	(17.9)		1,897	(19.9)		5,939	(22.1)
60–69		2,292	(13.2)		1,288	(13.5)		3,503	(13.1)
≥ 70		957	(5.5)		432	(4.5)		866	(3.2)
Sex									
Male		16,154	(93.3)		9,397	(98.7)		26,648	(99.3)
Female		1,151	(6.7)		126	(1.3)		186	(0.7)
State									
Iowa		8,583	(49.6)		6,754	(70.9)		19,780	(73.7)
North Carolina		8,722	(50.4)		2,769	(29.1)		7,054	(26.3)
Smoking status (pack-years)									
Never		8,580	(52.9)		5,205	(56.2)		14,408	(55.3)
Former (< 3.75)		1,583	(9.8)		998	(10.8)		2,844	(10.9)
Former (3.75–15 )		1,571	(9.7)		873	(9.4)		2,440	(9.4)
Former (> 15 )		1,524	(9.4)		800	(8.6)		2,339	(9.0)
Current (< 11.25)		1,283	(7.9)		475	(5.1)		1,312	(5.0)
Current (11.25–28.5)		1,055	(6.5)		456	(4.9)		1,319	(5.1)
Current (> 28.5)		1,031	(6.4)		450	(4.9)		1,412	(5.4)
Missing		678			266			760	
Alcohol use									
Never		6,230	(38.3)		2,836	(31.1)		7,387	(28.4)
Ever in last year		10,026	(61.7)		6,287	(68.9)		18,655	(71.6)
Missing		1,049			400			792	
Education									
< High school		2,143	(13.2)		836	(8.9)		1,896	(7.2)
High school graduate		7,511	(46.2)		4,496	(48.1)		12,976	(49.3)
> High school		7,200	(44.3)		4,011	(42.9)		11,456	(43.5)
Missing		451			180			506	
Family history of cancer									
No		10,031	(62.8)		5,297	(58.5)		14,774	(57.2)
Yes		5,954	(37.2)		3,750	(41.5)		11,070	(42.8)
Missing		1,320			476			990	
Applicator type									
Private		14,967	(86.5)		9,173	(96.3)		24,888	(92.7)
Commercial		2,338	(13.5)		350	(3.7)		1,946	(7.3)
Use of dicamba									
No		11,908	(76.4)		3,790	(43.5)		9,173	(36.2)
Yes		3,682	(23.6)		4,914	(56.5)		16,160	(63.8)
Missing		1,715			819			1,501	
Use of cyanazine									
No		13,922	(89.5)		5,064	(58.0)		10,290	(40.4)
Yes		1,625	(10.5)		3,673	(42.0)		15,188	(59.6)
Missing		1,758			786			1,356	
Use of metolachlor									
No		12,590	(81.1)		4,605	(52.6)		9,573	(37.5)
Yes		2,937	(18.9)		4,143	(47.4)		15,923	(62.5)
Missing		1,778			775			1,338	
Use of trifluralin									
No		11,369	(73.8)		3,784	(43.0)		8,626	(33.8)
Yes		4,029	(26.2)		5,006	(57.0)		16,910	(66.2)
Missing		1,907			733			1,298	
Use of 2,4-D									
No		8,165	(47.8)		1,892	(20.0)		3,597	(13.5)
Yes		8,901	(52.2)		7,554	(80.0)		23,056	(86.5)
Missing		239			77			181	

Overall, there were 4,737 incident cancers diagnosed among 53,662 applicators through 2007, including 1,785 who were diagnosed since the previous report on atrazine and cancer incidence from the AHS (Rusiecki et al. 2004). Among the 36,357 atrazine users, there were 3,146 incident cancer diagnoses. As shown in Table 2, no association for cancer overall with either the lifetime days or intensity-weighted metric was apparent. For most cancer sites, there was little evidence for an association between atrazine use and incident cancer, including cancers of the prostate and lung, two of the most common sites diagnosed in this cohort. In general, results for the intensity-weighted and lifetime days of use metrics were similar.

**Table 2:** Solid tumors among 36,357 atrazine users in the AHS, 1993–2007.

Lifetime days				Intensity-weighted lifetime days			
Cancer site	No. of cases	RR*a* (95% CI)		No. of cases*b*	RR*a* (95% CI)	
All										
Q1: > 0 to ≤ 20		832		1.00	Reference		786		1.00	Reference
Q2: > 20 to ≤ 56		771		1.03	(0.93–1.13)		785		0.99	(0.90–1.10)
Q3: > 56 to ≤ 178.5		834		1.00	(0.90–1.10)		785		1.03	(0.93–1.14)
Q4: > 178.5		709		1.06	(0.96–1.18)		785		1.01	(0.91–1.11)
				*p*-trend = 0.26					*p*-trend = 0.90	
Prostate										
Q1: > 0 to ≤ 20		335		1.00	Reference		316		1.00	Reference
Q2: > 20 to ≤ 56		317		1.05	(0.90–1.22)		331		1.04	(0.89–1.21)
Q3: > 56 to ≤ 178.5		357		1.03	(0.88–1.19)		326		1.06	(0.91–1.24)
Q4: > 178.5		288		1.06	(0.90–1.25)		322		1.04	(0.89–1.22)
				*p*-trend = 0.55					*p*-trend = 0.81	
Lung*c*										
Q1: > 0 to ≤ 20		84		1.00	Reference		65		1.00	Reference
Q2: > 20 to ≤ 56		64		0.89	(0.64–1.24)		69		1.03	(0.73–1.45)
Q3: > 56 to ≤ 178.5		69		0.86	(0.62–1.19)		69		1.03	(0.73–1.46)
Q4: > 178.5		58		0.86	(0.60–1.22)		72		0.93	(0.66–1.32)
				*p*-trend = 0.53					*p*-trend = 0.56	
Colon										
Q1: > 0 to ≤ 20		65		1.00	Reference		63		1.00	Reference
Q2: > 20 to ≤ 56		40		0.70	(0.47–1.04)		47		0.75	(0.51–1.09)
Q3: > 56 to ≤ 178.5		67		1.06	(0.75–1.50)		52		0.86	(0.59–1.25)
Q4: > 178.5		64		1.26	(0.88–1.80)		73		1.16	(0.82–1.65)
				*p*-trend = 0.04					*p*-trend = 0.07	
Rectum										
Q1: > 0 to ≤ 20		28		1.00	Reference		26		1.00	Reference
Q2: > 20 to ≤ 56		25		0.96	(0.56–1.64)		26		1.02	(0.59–1.76)
Q3: > 56 to ≤ 178.5		33		1.16	(0.69–1.94)		34		1.31	(0.78–2.20)
Q4: > 178.5		27		1.14	(0.66–1.96)		27		1.02	(0.59–1.78)
				*p*-trend = 0.60					*p*-trend = 0.94	
Bladder*c*										
Q1: > 0 to ≤ 20		47		1.00	Reference		41		1.00	Reference
Q2: > 20 to ≤ 56		33		0.79	(0.50–1.24)		34		0.82	(0.52–1.30)
Q3: > 56 to ≤ 178.5		31		0.68	(0.43–1.08)		35		0.88	(0.56–1.39)
Q4: > 178.5		30		0.84	(0.52–1.36)		31		0.75	(0.46–1.21)
				*p*-trend = 0.76					*p*-trend = 0.34	
Oral cavity										
Q1: > 0 to ≤ 20		14		1.00	Reference		17		1.00	Reference
Q2: > 20 to ≤ 56		27		2.09	(1.09–3.99)		18		1.10	(0.57–2.15)
Q3: > 56 to ≤ 178.5		20		1.43	(0.72–2.86)		21		1.33	(0.70–2.54)
Q4: > 178.5		13		1.14	(0.53–2.47)		18		1.20	(0.61–2.36)
				*p*-trend = 0.54					*p*-trend = 0.70	
Esophagus										
Q1: > 0 to ≤ 20		7		1.00	Reference		5		1.00	Reference
Q2: > 20 to ≤ 56		14		2.11	(0.85–5.25)		15		2.83	(1.03–7.82)
Q3: > 56 to ≤ 178.5		15		1.99	(0.80–4.96)		12		2.33	(0.81–6.67)
Q4: > 178.5		6		0.90	(0.29–2.74)		10		1.78	(0.59–5.30)
				*p*-trend = 0.40					*p*-trend = 0.74	
		Lifetime days					Intensity-weighted lifetime days			
Cancer site		No. of cases		RR*a* (95% CI)			No. of cases*b*		RR*a* (95% CI)	
Pancreas										
Q1: > 0 to ≤ 20		15		1.00	Reference		20		1.00	Reference
Q2: > 20 to ≤ 56		13		0.95	(0.45–2.02)		8		0.38	(0.17–0.87)
Q3: > 56 to ≤ 178.5		10		0.65	(0.29–1.46)		13		0.63	(0.31–1.28)
Q4: > 178.5		13		1.01	(0.46–2.19)		10		0.45	(0.21–0.98)
				*p*-trend = 0.86					*p*-trend = 0.18	
Larynx										
Q1: > 0 to ≤ 20		8		1.00	Reference		9		1.00	Reference
Q2: > 20 to ≤ 56		6		0.82	(0.28–2.38)		7		0.76	(0.28–2.04)
Q3: > 56 to ≤ 178.5		8		0.97	(0.35–2.65)		4		0.44	(0.13–1.45)
Q4: > 178.5		2		0.24	(0.05–1.18)		4		0.36	(0.11–1.21)
				*p*-trend = 0.05					*p*-trend = 0.09	
Cutaneous melanoma										
Q1: > 0 to ≤ 20		38		1.00	Reference		35		1.00	Reference
Q2: > 20 to ≤ 56		31		0.89	(0.55–1.43)		39		1.15	(0.73–1.82)
Q3: > 56 to ≤ 178.5		29		0.82	(0.50–1.35)		30		0.93	(0.57–1.53)
Q4: > 178.5		34		1.15	(0.71–1.87)		28		0.85	(0.51–1.42)
				*p*-trend = 0.36					*p*-trend = 0.33	
Kidney										
Q1: > 0 to ≤ 20		27		1.00	Reference		28		1.00	Reference
Q2: > 20 to ≤ 56		19		0.78	(0.43–1.41)		18		0.65	(0.36–1.17)
Q3: > 56 to ≤ 178.5		29		1.11	(0.65–1.88)		26		0.97	(0.57–1.67)
Q4: > 178.5		24		1.21	(0.69–2.13)		27		1.02	(0.59–1.76)
				*p*-trend = 0.31					*p*-trend = 0.47	
Brain										
Q1: > 0 to ≤ 20		9		1.00	Reference		9		1.00	Reference
Q2: > 20 to ≤ 56		9		0.99	(0.39–2.51)		10		1.08	(0.44–2.67)
Q3: > 56 to ≤ 178.5		10		1.01	(0.40–2.52)		11		1.16	(0.48–2.84)
Q4: > 178.5		8		0.97	(0.35–2.70)		6		0.62	(0.21–1.78)
				*p*-trend = 0.92					*p*-trend = 0.24	
Thyroid										
Q1: > 0 to ≤ 20		4		1.00	Reference		3		1.00	Reference
Q2: > 20 to ≤ 56		11		2.91	(0.92–9.21)		12		4.55	(1.27–16.24)
Q3: > 56 to ≤ 178.5		7		1.86	(0.53–6.51)		3		1.16	(0.23–5.80)
Q4: > 178.5		7		2.32	(0.66–8.22)		11		4.84	(1.31–17.93)
				*p*-trend = 0.61					*p*-trend = 0.08	
Liver										
Q1: > 0 to ≤ 20		6		1.00	Reference		5		1.00	Reference
Q2: > 20 to ≤ 56		8		1.34	(0.46–3.90)		9		1.65	(0.55–4.95)
Q3: > 56 to ≤ 178.5		3		0.47	(0.12–1.94)		3		0.54	(0.13–2.28)
Q4: > 178.5		11		2.13	(0.76–5.99)		11		1.84	(0.62–5.48)
				*p*-trend = 0.09					*p*-trend = 0.27	
Q, quartile. **a**Controlled for age, state, license type, sex, smoking (never, ≤ 12 pack years, > 12 pack-years), alcohol consumption, education, and use of most highly correlated pesticides. **b**Number of cases differ between the two metrics because of missing data for intensity-weighting factors. **c**Additionally controlled for smoking as never and tertiles of former and current smoking.

Based on 29 exposed cases, there was a statistically significant association between thyroid cancer and atrazine intensity-weighted lifetime days of use in the highest quartile of use [RR = 4.84; 95% confidence interval (CI), 1.31–17.93, *p*-trend = 0.08] (Table 2). For lifetime days of use, there was also a suggestion of an association in the highest category (RR = 2.32; 95% CI, 0.66–8.22, *p*-trend = 0.61). However, neither metric exhibited a monotonic trend. Because obesity has been linked with increased risk of thyroid cancer (Kitahara et al. 2011), we further adjusted these results for body mass index. The RRs for intensity-weighted lifetime days were 4.30 (95% CI, 1.21–15.28), 1.09 (95% CI, 0.22–5.43), and 4.30 (95% CI, 1.19–15.57) for the second, third, and fourth quartiles of exposure, respectively, compared with the lowest quartile (*p*-trend = 0.30). We were not able to stratify results by applicator type, because only one case of thyroid cancer occurred in commercial applicators and removing this case did not change risk estimates (data not shown). Twenty-two of the 29 exposed cases occurred in Iowa, and the RRs were 3.88 (95% CI, 1.01–14.90), 0.87 (95% CI, 0.14–5.42), and 4.50 (95% CI, 1.12–18.15) for the second, third, and fourth quartiles. There were no cases in North Carolina in the lowest quartile of exposure.

Esophageal cancer was significantly elevated in the second category of exposure for intensity-weighted lifetime days (RR = 2.83; 95% CI, 1.03–7.82), but not in the higher categories of intensity-weighted lifetime days (RR = 2.33; 95% CI, 0.81–6.67 and RR = 1.78; 95% CI, 0.59–5.30) or for lifetime days (Table 2). Similarly, a statistically significant increased risk for cancer of the oral cavity in the second category of atrazine lifetime use compared with the lowest use category was observed (RR = 2.09; 95% CI, 1.09–3.99). However, the RRs in the higher two quartiles were closer to the null (RR = 1.43 and 1.19, respectively), and RRs for intensity-weighted lifetime days were not significantly elevated. RRs for intensity-weighted lifetime days were not elevated. For pancreatic cancer (*n* = 51), there were decreased risks in all exposure categories, which was statistically significant in the second exposure category for intensity-weighted lifetime days (RR = 0.38; 95% CI, 0.17–0.87, *p*-trend = 0.18), but no evidence of any association with lifetime days of exposure. Based on 24 cases, there were decreased risks for laryngeal cancer for both metrics of exposure in the second, third, and fourth quartiles, although none were statistically significant (*p*-trend = 0.05 and *p*-trend = 0.09 for lifetime and intensity-weighted lifetime days, respectively).

Because of *a priori* interest in hormonally related cancers, we also evaluated use of atrazine and breast cancer (*n* = 36) and ovarian cancer (*n* = 9) among female pesticide applicators, although the number of cases was limited. We used the nonexposed category as the referent (*n* = 1,154) and evaluated only ever use of atrazine (*n* = 312). Results presented are adjusted only for age, but results adjusted for other factors, including parity, were similar (data not shown). There was no association between atrazine use and breast cancer based on 9 exposed and 27 unexposed cases (RR = 1.14; 95% CI, 0.47–2.50). Assessing risk for those above the median level of lifetime days of use also provided no evidence of risk. There were only four cases of breast cancer among male applicators, which precluded us from carrying out a separate analysis for breast cancer among men. For ovarian cancer, the RR for ever use of atrazine was 2.91 (95% CI, 0.56–13.60), based on four cases among the women who reported atrazine use and five cases among those who reported no use. All exposed cases occurred among those with less than the median of atrazine use among all cancer cases.

Use of atrazine did not appear to be associated with any lymphohematopoietic malignancy (Table 3). For NHL, RRs were near 1.0 for both lifetime days and intensity-weighted lifetime days. Similarly, null patterns were observed for all subtypes. We evaluated potential effect modification by use of chemicals previously reported to interact with atrazine and NHL (alachlor, carbofuran, and diazinon) by conducting analyses stratified by use of these chemicals. We saw no evidence of stratum-specific differences (data not shown). There was also no association with leukemia or multiple myeloma.

**Table 3:** Lymphohematopoietic malignancies among 36,357 atrazine users in the AHS, 1993–2007.

Lifetime days				Intensity-weighted lifetime days			
No. of cases	RR*a* (95% CI)		No. of cases*b*	RR*a* (95% CI)	
Lymphohematopoietic malignancies										
Q1: > 0 to ≤ 20		81		1.00	Reference		81		1.00	Reference
Q2: > 20 to ≤ 56		84		1.12	(0.82–1.52)		84		1.04	(0.76–1.41)
Q3: > 56 to ≤ 178.5		82		0.97	(0.71–1.33)		78		0.98	(0.71–1.34)
Q4: > 178.5		64		0.94	(0.67–1.32)		66		0.83	(0.59–1.16)
				*p*-trend = 0.49					*p*-trend = 0.17	
Leukemia										
Q1: > 0 to ≤ 20		23		1.00	Reference		27		1.00	Reference
Q2: > 20 to ≤ 56		31		1.41	(0.82–2.43)		26		0.96	(0.56–1.64)
Q3: > 56 to ≤ 178.5		24		0.97	(0.54–1.73)		24		0.91	(0.52–1.58)
Q4: > 178.5		19		0.95	(0.51–1.79)		19		0.72	(0.39–1.31)
				*p*-trend = 0.46					*p*-trend = 0.24	
NHL										
Q1: > 0 to ≤ 20		41		1.00	Reference		38		1.00	Reference
Q2: > 20 to ≤ 56		41		1.10	(0.71–1.70)		45		1.20	(0.78–1.86)
Q3: > 56 to ≤ 178.5		38		0.92	(0.59–1.45)		34		0.93	(0.58–1.48)
Q4: > 178.5		32		0.96	(0.60–1.55)		34		0.93	(0.58–1.50)
				*p*-trend = 0.73					*p*-trend = 0.48	
Mature B-cell lymphoma										
Q1: > 0 to ≤ 20		36		1.00	Reference		34		1.00	Reference
Q2: > 20 to ≤ 56		34		1.04	(0.65–1.66)		38		1.12	(0.71–1.79)
Q3: > 56 to ≤ 178.5		31		0.85	(0.52–1.38)		25		0.75	(0.45–1.27)
Q4: > 178.5		28		0.94	(0.57–1.57)		31		0.93	(0.57–1.54)
				*p*-trend = 0.79					*p*-trend = 0.65	
Diffuse large B-cell lymphoma										
Q1: > 0 to ≤ 20		20		1.00	Reference		15		1.00	Reference
Q2: > 20 to ≤ 56		14		0.80	(0.40–1.59)		18		1.23	(0.62–2.45)
Q3: > 56 to ≤ 178.5		14		0.73	(0.36–1.47)		11		0.78	(0.35–1.71)
Q4: > 178.5		11		0.73	(0.34–1.57)		15		1.09	(0.52–2.27)
				*p*-trend = 0.52					*p*-trend = 0.96	
Follicular lymphoma										
Q1: > 0 to ≤ 20		10		1.00	Reference		10		1.00	Reference
Q2: > 20 to ≤ 56		8		0.85	(0.33–2.17)		10		0.99	(0.41–2.38)
Q3: > 56 to ≤ 178.5		6		0.60	(0.22–1.70)		8		0.80	(0.31–2.05)
Q4: > 178.5		8		0.99	(0.38–2.59)		4		0.39	(0.12–1.28)
				*p*-trend = 0.85					*p*-trend = 0.07	
CLL/SLL/MCL										
Q1: > 0 to ≤ 20		5		1.00	Reference		6		1.00	Reference
Q2: > 20 to ≤ 56		6		1.23	(0.37–4.03)		6		0.96	(0.31–3.00)
Q3: > 56 to ≤ 178.5		11		1.87	(0.64–5.47)		4		0.66	(0.18–2.37)
Q4: > 178.5		4		0.77	(0.20–3.00)		9		1.44	(0.49–4.23)
				*p*-trend = 0.47					*p*-trend = 0.33	
Multiple myeloma										
Q1: > 0 to ≤ 20		14		1.00	Reference		13		1.00	Reference
Q2: > 20 to ≤ 56		9		0.71	(0.31–1.64)		10		0.71	(0.31–1.63)
Q3: > 56 to ≤ 178.5		13		0.83	(0.38–1.79)		14		0.98	(0.45–2.10)
Q4: > 178.5		13		1.04	(0.47–2.29)		12		0.79	(0.35–1.76)
				*p*-trend = 0.45					*p*-trend = 0.87	
Abbreviations: CLL, chronic lymphocytic leukemia; SLL, small lymphocytic lymphoma; MCL, mantle cell lymphoma; Q, quartile. **a**Controlled for age, state, license type, sex, smoking (never, ≤ 12 pack years, > 12 pack-years), alcohol consumption, education and use of most highly correlated pesticides. **b**Number of cases differ between the two metrics because of missing data for intensity-weighting factors.										

Because of the differences in pesticide usage patterns between private and commercial applicators and the higher prevalence of atrazine usage in Iowa than in North Carolina, we also evaluated each cancer site stratified by state and by license type (private vs. commercial). We observed no meaningful differences in the results for any cancer site (data not shown).

## Discussion

There was no association observed between use of atrazine and cancer overall and no evidence for an association with most cancer sites. However, there was limited evidence for an association with atrazine use and cancer of the thyroid and ovaries.

Atrazine is an endocrine-disrupting chemical. It has been hypothesized that exposure to atrazine may alter hormone levels, possibly through induction of aromatase, which converts androgens into estrogens (Gammon et al. 2005). Atrazine causes mammary tumors in rats by affecting the hypothalamus, which affects the pituitary gland and ultimately disrupts luteinizing hormone cycling, leading to increases in endogenous estrogen and prolactin (Gammon et al. 2005). In rats, this altered exposure to reproductive hormones produces accelerated reproductive aging and a hormonal environment that is conducive to mammary tumor development. This mechanism does not appear to operate in humans. However, the hypothalamic regulation of luteinizing hormone and prolactin secretion is similar, suggesting that atrazine may influence pituitary hormones (Gammon et al. 2005). In addition, inhibition of luteinizing hormone in male rats has been linked to reduction of testosterone (Trentacoste et al. 2001).

We observed a non–statistically significant increased risk of ovarian cancer among females who ever used atrazine; however, this observation is based on only four exposed cases. Because of the small number of cases, we were able to evaluate only an association for ever using atrazine and not any potential exposure–response relationships, which is a limitation of this analysis. We cannot rule out chance as an explanation for this finding, even though we explored this association based on *a priori* interest because of the hormonal properties of atrazine. Uncontrolled confounding could also explain these results. We previously reported an excess risk of ovarian cancer incidence and mortality among all female pesticide applicators in the AHS compared with the general population of Iowa and North Carolina (Alavanja et al. 2005; Koutros et al. 2010; Waggoner et al. 2010). The absence of an excess for ovarian cancer among the approximately 32,000 women married to pesticide applicators but who are not themselves licensed applicators suggests that either pesticides or other occupational factors may contribute to the excess among female pesticide applicators. There is a paucity of epidemiologic literature on ovarian cancer and atrazine. Case–control studies in Italy and California of ovarian cancer and triazine exposure, the chemical group to which atrazine belongs, were suggestive of an association, but neither was statistically significant (Donna et al. 1989; Young et al. 2005). An ecologic study showed an inverse association between district-level exposure metrics (which included public drinking water atrazine levels, acreage of corn planted, and sales of atrazine) and ovarian cancer (Hopenhayn-Rich et al. 2002).

Compared with nonusers, there was little evidence of an increased risk of breast cancer among female pesticide applicators who reported use of atrazine, based on nine exposed cases. Data from other epidemiologic studies of breast cancer and atrazine are limited. An earlier study within the AHS that included wives of pesticide applicators reported no association between breast cancer and atrazine use, whether use was classified by personal use or use by the husband (Engel et al. 2005). Ecologic studies have generally not suggested such associations (Hopenhayn-Rich et al. 2002; Mills and Yang 2006), although one study in England observed a spatial association between breast cancer incidence rates and the application of atrazine in one county, but not another (Muir et al. 2004). The IARC classification for atrazine is Group 3 (not classifiable), but the Working Group determined that there was sufficient evidence for carcinogenicity in animals because of the association with mammary tumors. However, they determined that the atrazine-associated mammary tumors observed in rats involved a hormonally mediated mechanism not relevant for humans (IARC 1999).

Prostate cancer is a site hypothesized to be potentially associated with atrazine because of its hormonal etiology. We saw no evidence of an association with atrazine use and prostate cancer in this study, which is consistent with earlier results from the AHS (Alavanja et al. 2003; Rusiecki et al. 2004). In a study of pesticide manufacturing workers, there was an increased risk of prostate cancer, but this population had a very high rate of prostate cancer screening and 9 of the 11 cases were diagnosed at an early stage (MacLennan et al. 2002), suggesting that detection bias might be partially responsible for the observed results.

We found some evidence for a positive association between atrazine use and thyroid cancer in this analysis. The epidemiologic evidence on pesticides and thyroid cancer is scarce. A report from Yugoslavia suggested an association with agricultural chemicals (Sokic et al. 1994), but no specific pesticides were implicated. A cohort of female farm residents in New York indicated a nonsignificant increased standardized incidence ratio for thyroid cancer, but without evaluation of specific exposures (Wang et al. 2002). The previous report from the AHS on cancer incidence and atrazine did not include thyroid cancer, because there were < 20 exposed cases, the cutoff for presenting results in the earlier paper (Rusiecki et al. 2004); with this update, there were 29 cases exposed to atrazine. Thyroid cancer is more common among women than men; in this study of primarily male applicators, there was only one female case among 312 atrazine users compared with 28 among the 36,045 male applicators who applied atrazine. Hyperthyroidism has been linked to thyroid cancer in some studies; however, in a study among spouses of applicators in the AHS who applied pesticides, there was no association between use of atrazine and this condition (Goldner et al. 2010). Endocrine-disrupting substances can disrupt hormonal synthesis or metabolism and may impact the thyroid function in a variety of ways (Diamanti-Kandarakis et al. 2009). However, studies based on bioassays specific for atrazine have been generally null (Hasegawa et al. 1993), and studies in rodents have shown limited effects of atrazine on the thyroid (Laws et al. 2000; Son et al. 2003). We found a significantly elevated risk for the highest versus lowest category of intensity-weighted atrazine use, but the findings were based on a relatively small number of exposed cases, and the trend was not monotonic. We know of no other reports that have investigated atrazine and thyroid cancer, and this association needs further evaluation.

There is some epidemiologic evidence for an association between atrazine exposure and NHL. Three early case–control studies focused on triazine herbicides and showed non–statistically significant increased NHL risk (Cantor et al. 1992; Hoar et al. 1986; Zahm et al. 1993). NHL was more common among those who used atrazine compared with those who had never worked on a farm, based on a pooled analysis of these studies (Hoar Zahm et al. 1993); further analysis of these pooled data included evidence of supra-additive effects when atrazine was considered in conjunction with other pesticides, namely, carbofuran, diazinon, and alachlor (De Roos et al. 2003). We saw no such evidence in this analysis. In the previous analysis of atrazine within the AHS, there was a nonsignificant excess reported with NHL based on 68 exposed cases (Rusiecki et al. 2004). Here, we observed 152 exposed cases and no evidence of association. Other studies of atrazine and NHL include a mortality study of pesticide manufacturing workers that showed a non–statistically significant excess of NHL based on four deaths (MacLennan et al. 2003). Schroeder et al. (2001) evaluated associations with agricultural exposures by t(14;18) subtypes and reported that atrazine was associated with NHL among those cases with the translocation, but not among those without. The translocation was most common in follicular (46%), diffuse B cell (39%), other (66%), and unclassified (52%) subtypes. In our study, we did not have tumor tissue to evaluate such an association but were able to look at histologic subtypes based on cancer registry records and saw no association for any subtype.

We found no association with multiple myeloma with either lifetime days of use or intensity-weighted lifetime days of use. In the previous AHS analysis of atrazine, there was a suggestion of an association based on 23 exposed cases (Rusiecki et al. 2004). Our analysis includes an additional 26 exposed cases for a total of 49. The only other study specifically evaluating multiple myeloma showed little evidence for such an association (Brown et al. 1993).

A previous analysis of atrazine use in the AHS also suggested non–statistically significant associations with cancers of the bladder and lung (Rusiecki et al. 2004). In this update, with more than twice as many cases for these sites, we observed no evidence of association. To our knowledge, no other studies support an association at these sites.

Strengths of this study include the collection of information on atrazine use before the diagnosis of cancer, the relatively large number of exposed cases, and the ability to control for potential confounding by use of other agricultural chemicals. Although this is a large cohort study, there are still few female applicators, limiting the power to evaluate female-specific cancers such as breast and ovarian cancer, which have been hypothesized to be associated with atrazine. Additionally, we used self-reported information on the use of atrazine, the methods of application, and the use of personal protective equipment. Although this is a limitation, the reliability of the data on use of pesticides within this cohort is comparable with or more reliable than other self-reported data such as diet and medical conditions (Blair et al. 2002), and pesticide applicators in the AHS report the duration and time of first use of specific chemicals consistent with the time that the chemicals first became available (Hoppin et al. 2002).

## Conclusion

In summary, there was no strong or consistent evidence of an association between atrazine and any cancer. There was a non–statistically significant increased risk of ovarian cancer among female applicators who reported ever using atrazine compared with those who did not; however, this observation was based on a small number of cases among atrazine users (*n* = 4). We found an elevated risk of thyroid cancer for the highest versus lowest category of intensity-weighted atrazine use, but the trend was not monotonic and not statistically significant when lifetime days of use was used as the exposure metric. An association between atrazine and thyroid cancer has not been previously reported, to our knowledge. In contrast, we saw little evidence for an association between atrazine use and other cancers previously reported in the literature, such as NHL and leukemia, or with cancers of the breast or prostate, for which atrazine has been hypothesized to be a risk factor because of its hormonal properties.
